# Morphological Effects of Au Nanoparticles on Electrochemical Sensing Platforms for Nitrite Detection

**DOI:** 10.3390/molecules28134934

**Published:** 2023-06-23

**Authors:** Ruiqin Feng, Ye Fan, Yun Fang, Yongmei Xia

**Affiliations:** 1 The Key Laboratory of Synthetic and Biological Colloids (Ministry of Education), School of Chemical and Material Engineering, Jiangnan University, Wuxi 214122, China; 7170610001@stu.jiangnan.edu.cn (R.F.);; 2 State Key Laboratory of Food Science and Technology, Jiangnan University, Wuxi 214122, China

**Keywords:** Au nanoparticle, Au/GCE, nitrite, cyclic voltammetry, polyvinylpyrrolidone, sodium lauryl sulfate

## Abstract

Au nanoparticles were synthesized in a soft template of pseudo-polyanions composed of polyvinylpyrrolidone (PVP) and sodium dodecyl sulfate (SDS) by the in situ reduction of chloroauric acid (HAuCl_4_) with PVP. The particle sizes and morphologies of the Au nanoparticles were regulated with concentrations of PVP or SDS at room temperature. Distinguished from the Au nanoparticles with various shapes, Au nanoflowers (AuNFs) with rich protrusion on the surface were obtained at the low final concentration of SDS and PVP. The typical AuNF synthesized in the PVP (50 g·L^−1^)–SDS (5 mmol·L^−1^)–HAuCl_4_ (0.25 mmol·L^−1^) solution exhibited a face-centered cubic structure dominated by a {111} crystal plane with an average equivalent particle size of 197 nm and an average protrusion height of 19 nm. Au nanoparticles with four different shapes, nanodendritic, nanoflower, 2D nanoflower, and nanoplate, were synthesized and used to modify the bare glassy carbon electrode (GCE) to obtain Au/GCEs, which were assigned as AuND/GCE, AuNF/GCE, 2D-AuNF/GCE, and AuNP/GCE, respectively. Electrochemical sensing platforms for nitrite detection were constructed by these Au/GCEs, which presented different detection sensitivity for nitrites. The results of cyclic voltammetry (CV) demonstrated that the AuNF/GCE exhibited the best detection sensitivity for nitrites, and the surface area of the AuNF/GCE was 1.838 times of the bare GCE, providing a linear *c*(NO_2_^−^) detection range of 0.01–5.00 µmol·L^−1^ with a limit of detection of 0.01 µmol·L^−1^. In addition, the AuNF/GCE exhibited good reproducibility, stability, and high anti-interference, providing potential for application in electrochemical sensing platforms.

## 1. Introduction

Nitrites are often used in food for color protection and as preservatives [1], although excessive and long-term intake of nitrites may cause dizziness, cancer, and fetal deformity [1,2,3]. Therefore, it is important to strictly monitor the nitrite content with high sensitivity in a wide detection range.

The present nitrite detection methods include ultraviolet spectrophotometry [4], chromatography [5], chromatography-mass spectrometry [6], fluorescence [7], electrochemical methods [8], biosensors [9], and colorimetric methods [10]. These detection methods have advantages of high sensitivity, fast response, and high accuracy; but most of them involve complicated sample preparation, expensive instruments, and time-consuming operations [5,6,9,11]. Electrochemical sensing platforms that involve the electrocatalytic oxidation of nitrites are widely applied in nitrite detection due to the advantages of high selectivity, fast response speed, portability, field inspection, and easy operation [12,13,14], but the electrocatalytic oxidation of nitrites detected by glassy carbon electrodes (GCEs) usually has a high overpotential [15,16,17]. To eliminate the overpotential issue, the modification of the GCE surface with nanoparticles has been applied, which can enhance its adsorption capacity and conductivity and provide more catalytic active sites to improve the detection sensitivity of GCE [14,15,17]. The nano/submicron materials of metals [18], metal oxides [14], conductive polymers [19], and carbon and its derivatives [20,21,22] are used to modify GCE; among them, Au nanoparticles have become a popular choice for GCE modification due to the chemical stability, biocompatibility, good conductivity, and electrocatalytic activity of gold [11], and they are often used in combination with other metals [23], graphene, MOFs [12] or polymers [24] to improve the detection sensitivity of GCE. However, the modification process of GCE often requires multiple steps, and the particle sizes and morphologies of the Au nanostructure are difficult to control.

Gold nanoflowers (AuNFs) have attracted more attention because of the large specific surface area and the resultant high catalytic or SERS activities [25,26]. Researchers used to synthesize the AuNFs by seed-mediated growth and template protection [27,28]. However, with the seed-mediated growth method, it is difficult to control the particle sizes and morphologies of the Au particles that require further loading on the substrate surface to form nano-protrusion. However, with the template protection method, strong adsorption of the commonly used cationic template materials may seriously interfere with the subsequent application of the as-synthesized Au nanoparticles. Therefore, anionic template materials composed of polyvinylpyrrolidone (PVP) and sodium lauryl sulfate (SDS) were used in this work instead of cationic surfactants. The AuNFs together with other morphology-controllable Au nanoparticles were synthesized in a one-pot process at room temperature, and the soft template function of the PVP–SDS pseudo-polyanions regarding the particle sizes and morphologies of the Au nanoparticles was investigated by regulating the concentrations of PVP or SDS. Furthermore, GCE was modified with the as-synthesized Au nanoparticles to construct electrochemical sensing platforms for nitrite detection, and the electrochemical behavior of NO_2_^−^ detected by the modified GCE was studied to examine the morphological effects of Au nanoparticles on the electrochemical sensing platforms for nitrite detection.

## 2. Results

### 2.1. Characterization of the Crystal Structures, Particle Sizes, and Morphologies of Au Nanoparticles

PVP has been used as stabilizing agent to form spherical nanoparticles via in situ reduction [29], or to yield ginger-like Au nanobranches in PVP–SDS aqueous solution under microwave irradiation [30].

**Figure 1:**
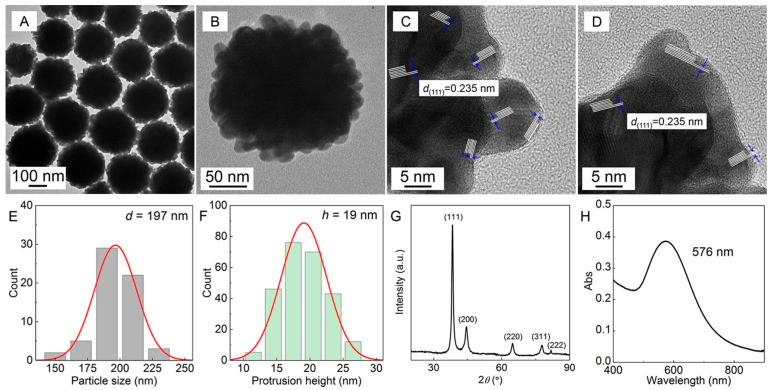
AuNFs synthesized in PVP (50 g∙L^−1^)–SDS (5 mmol·L^−1^)–HAuCl_4_ (0.25 mmol·L^−1^) solution characterized by (**A**,**B**) TEM, (**C**,**D**) HRTEM, (**E**) size distribution, (**F**) nano-protrusion height distribution, (**G**) XRD, and (**H**) LSPR.

With PVP (50 g∙L^−1^)–SDS (5 mmol·L^−1^) pseudo-polyanions as the soft template, typical Au nanoflowers (AuNFs) were synthesized, and their morphology, size, size distribution, XRD, and local surface plasmon resonance (LSPR) profiles are shown in Figure 1. The TEM, HRTEM, and particle size distribution of the AuNFs in Figure 1A–F show that the average equivalent particle size is 197 nm, the average height of nano-protrusion is 19 nm, and the main lattice fringe spacing is 0.235 nm. The diffraction peaks of the XRD pattern of Au in Figure 1G are consistent with the Au powder diffraction standard (JCPDS, 04-0784), indicating that the as-synthesized nanoparticles have a face-centered cubic Au nanostructure with a dominant {111} crystal plane, which is also consistent with the lattice information disclosed by the HRTEM profiles in Figure 1C−D.

The size of the initial Au crystal calculated by the Debye–Scherrer equation is 9.0 nm, which is similar to the size of the PVP-bound SDS micelles formed in the PVP–SDS pseudo-polyanions [31], suggesting that the PVP-bound SDS micelles play a role as a primary template to confine the size of the initial Au crystal. Figure 1H shows that the nano-protrusion structure and nano-size of the AuNFs cause an obvious LSPR characteristic peak at 576 nm.

Subsequently, possible correspondence between the morphology of the Au nanoparticles and the structure of the PVP–SDS pseudo-polyanion was verified.

### 2.2. The Effects of PVP–SDS Soft Templates on the Growth of Au Nanoparticles

First, the structure of the PVP–SDS pseudo-polyanion depends on the PVP-bound SDS micelles that were linked with PVP chains through cation bridging association [32,33,34]. When PVP-SDS was used as a soft template, the particle sizes and morphologies of the as-synthesized metallic nanoparticles would be affected by the soft template structure [35]. Therefore, the single or synergistic soft template effects of PVP and SDS on the synthesis of the Au nanoparticles are studied to verify how the particle sizes and morphologies of the as-synthesized Au nanoparticles are regulated by dint of the cation-bridging structure of the PVP–SDS pseudo-polyanions. In this work, 2 mmol·L^−1^ *c*(NaOH) and 0.25 mmol·L^−1^ *c*(HAuCl_4_) were adopted because higher *c*(NaOH) or *c*(HAuCl_4_) (Figure S1) would result in dominant Au nanoplates instead of other morphologies.

**Figure 2:**
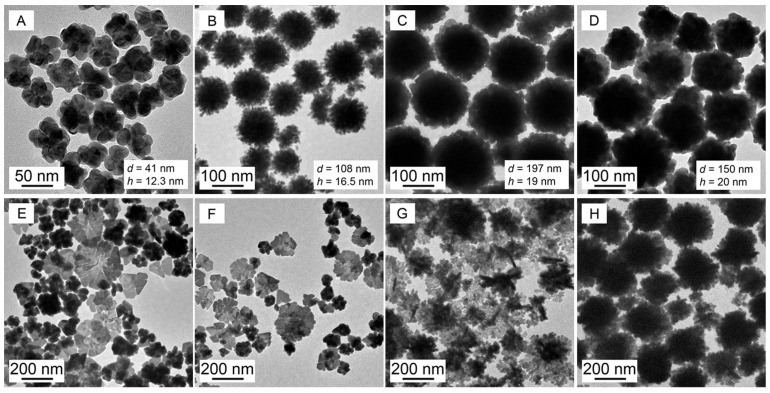
TEM images of Au nanoparticles growing in PVP (50 g·L^−1^)–SDS–HAuCl_4_ (0.25 mmol·L^−1^) solutions with *c*(SDS) at (**A**–**H**) 0, 2, 5, 15, 30, 45, 50 and 60 mmol·L^−1^. The data of *d* and *h* present the particle size and the protrusion height, respectively.

Au nanoparticles were synthesized in the above soft templates with a fixed *c*(PVP) and ever-increasing *c*(SDS); their TEM images are shown in Figure 2 with the corresponding LSPR spectra in Figure S2.

As a control by solely using PVP as both a reductant and a soft template, the obtained Au nanoparticles in Figure 2A were mainly irregular nanospheroids with an average equivalent particle size of 41 nm and without obvious nano-protrusion on the surface, and thus they were simply stacked by a few of the larger primary Au nanocrystals due to the lack of the confinement effects of the primary template provided by the PVP-bound SDS micelles on the growth of the primary Au crystals in the absence of SDS.

The AuNFs in Figure 2B–D were obtained in the *c*(SDS) range of 2–15 mmol·L^−1^ that is within the dual-critical concentration range (*c*_1_ and *c*_2_) of SDS in the presence of PVP [32,33]. The AuNFs possessed an average equivalent particle size range of 100–200 nm with rich 16–20 nm protrusion on the particle surface.

The above experimental results not only support the speculation that the PVP-bound SDS micelles serve as the primary template to confine the primary Au crystals similar to the micellar size in Figure 1 but also preliminarily reveal that PVP may display a secondary template to induce the formation of AuNFs. The PVP chain promotes the accumulation and stack of the controlled primary Au crystals in a finite space through selective adsorption and causes the continuous reduction of HAuCl_4_ on the preferred adsorbed {111} crystal plane of the primary Au crystals; both actions lead to the formation of AuNFs with increased particle size and surface protrusion. On the contrary, the Au nanoplates dominant in Figure 2E–G possess higher *c*(SDS), and even the 2D AuNFs (Figure 2H) correspond to the highest *c*(SDS) at 60 mmol·L^−1^. This is because excessive PVP-bound SDS micelles associated with the PVP chains weakened the reduction power of PVP, accelerating the nucleation of Au particles while slowing down the growth rate of the primary Au crystals and leading to the reduction process dominated by the crystal growth rule instead of the soft template control. 

Therefore, the above experimental results show that SDS is important for controlling the synthesis of AuNFs because only adequate *c*(SDS) can induce the formation of AuNFs and regulate both the particle size and the protrusion height of AuNFs or otherwise form either Au nanospheroids in the absence of SDS or Au nanoplates and 2D AuNFs in response to higher *c*(SDS).

**Figure 3:**
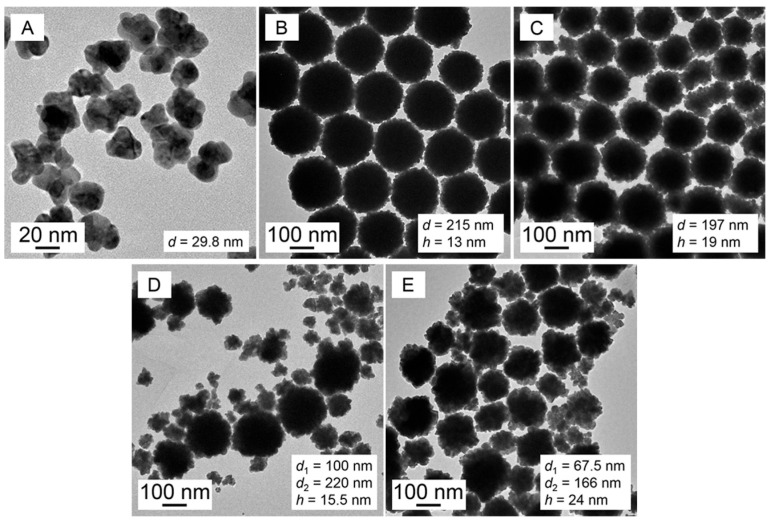
TEM images of Au nanoparticles growing in PVP–SDS (5 mmol·L^−1^)–HAuCl_4_ (0.25 mmol·L^−1^) solutions with *c*(PVP) at (**A**–**E**) 10, 30, 50, 80 and 100 g·L^−1^.

Subsequently, with a fixed *c*(SDS) and ever-increasing *c*(PVP), the Au nanoparticles were synthesized in SDS–PVP solutions (TEM, Figure 3; LSPR spectra, Figure S3).

Herein, PVP was the only reductant in the solution, so it is indispensable. The ever-increasing *c*(PVP) means increasing reduction power and thus accelerating growth rates of the primary Au crystals. The AuNFs exhibited the most obvious protrusion at a *c*(PVP) of 50 g∙L^−1^, indicating that for the formation of the AuNFs with obvious nano-protrusion, the best synergy is balanced among the reduction rate of HAuCl_4_, the competition between nucleation and the crystal growth of the gold particles, and the stacking degree of the primary Au crystals affected by *c*(PVP). Overhigh *c*(PVP) (>50 g∙L^−1^) would lead to too fast a reduction rate to be controlled, resulting in a lack of control of the particle sizes of Au nanoparticles and the presentation of a bimodal particle size distribution; in addition, the surface protrusion of the Au nanoparticles tends to be flat due to the Ostwald ripening effect. 

Therefore, PVP acted as not only the in situ reductant in the primary template but also as the secondary template to regulate the stacking degree of the primary Au crystals to fabricate AuNFs.

The next, the as-synthesized AuNFs are used to modify the bare GCE for nitrite detection based on the electrocatalytic oxidation of NO_2_^−^.

### 2.3. Electrocatalytic Oxidation of Nitrites Detected by Au/GCEs

#### 2.3.1. The Electrochemical Behavior of NO_2_^−^

The oxidation reaction of nitrites on Au/GCEs is irreversible (Equations (1) and (2)), and the corresponding reaction mechanism [8,11,36] is shown in Equation (3).

(1) $${\mathrm{NO}}_{2}^{-}\overset{\mathrm{Au}}{\to }{\mathrm{NO}}_{2}^{}+e^{-}$$

(2) $${2\mathrm{NO}}_{2}+{2H}_{2}O\overset{}{\to }{\mathrm{NO}}_{3}^{-}+{\mathrm{NO}}_{2}^{-}+{2H}^{+}$$

(3) $${\mathrm{NO}}_{2}^{-}+H_{2}O\overset{\mathrm{Au}}{\to }{\mathrm{NO}}_{3}^{-}+{2H}^{+}+{2e}^{-}$$

**Figure 4:**
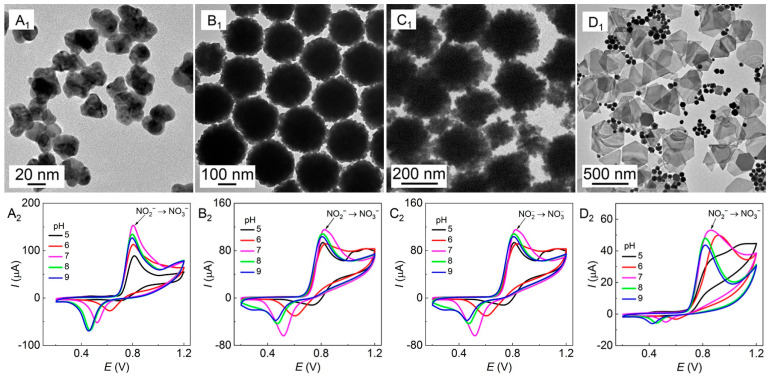
TEM images (**A_1_**–**D_1_**) of the Au nanoparticles growing in PVP–SDS–HAuCl_4_ and the CV plots (**A_2_**–**D_2_**) referring to the electrocatalytic oxidation of nitrites (5 mmol·L^−1^) detected by the corresponding Au/GCE at different pH values. A scan rate of 50 mV∙s^−1^ and 10 scanning turns; (**A**) the AuND/GCE in PVP (10 g·L^−1^)–SDS (5 mmol·L^−1^)–HAuCl_4_ (0.25 mmol·L^−1^), (**B**) the AuNF/GCE in PVP (50 g·L^−1^)–SDS (5 mmol·L^−1^)–HAuCl_4_ (0.25 mmol·L^−1^), (**C**) the 2D-AuNF/GCE in PVP (50 g·L^−1^)–SDS (60 mmol·L^−1^)–HAuCl_4_ (0.25 mmol·L^−1^), and (**D**) the AuNP/GCE in PVP (50 g·L^−1^)–SDS (5 mmol·L^−1^)–HAuCl_4_ (1.0 mmol·L^−1^).

The four Au nanoparticles with different shapes (Figure 4A_1_–D_1_, nanodendritic, nanoflower, 2D nanoflower, and nanoplate) were used to modify the bare GCE to obtain Au/GCEs (named AuND/GCE, AuNF/GCE, 2D-AuNF/GCE, and AuNP/GCE, respectively) to construct electrochemical sensing platforms for nitrite detection. Some of them were presented in Figure 2 and Figure 3 for comparison as well. The electrochemical behavior of NO_2_^−^ was detected with the four Au/GCEs at pH 5–9, as the cyclic voltammetry (CV) plots show in Figure 4A_2_–D_2_.

**Figure 5:**
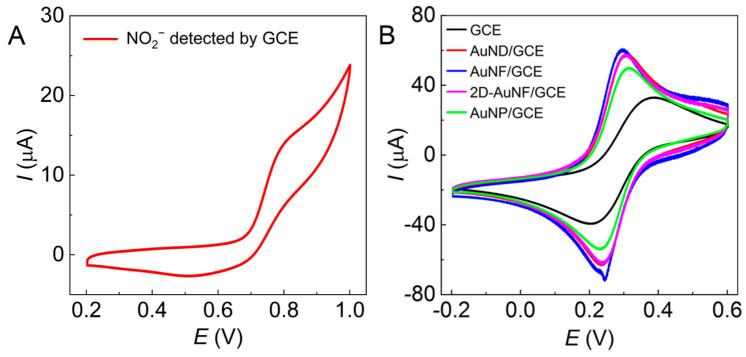
CV plots of (**A**) NO_2_^−^ (5 mmol·L^−1^) detected with the bare GCE and (**B**) the mixed solution of K_3_[Fe(CN)_6_] (5 mmol·L^−1^) and KCl (1 mol·L^−1^) detected by various Au/GCEs with a scan rate of 50 mV∙s^−1^ and 10 scanning turns.

With unmodified GCE, NO_2_^−^ at 5 mmol·L^−1^ (pH 7) induced an oxidation shoulder peak at 0.820 V with a corresponding redox potential difference of 0.310 V on the CV plots (Figure 5A), and the oxidation peak current (*I*_pa_) was only 15 µA, which was too low to determine the electrochemical behavior of NO_2_^−^. However, on all tested Au/GCEs, as shown in Figure 4A_2_–D_2_, NO_2_^−^ (5 mmol·L^−1^ at pH 5–9) provided an obvious sharp oxidation peak with a narrow oxidation peak potential (*E*_pa_) within 0.800–0.820 V except in Figure 4D_2_, which shows an undesired wide potential variation of 0.810–0.920 V. Meanwhile, the *I*_pa_ values in Figure 4A_2_–C_2_ are as high as 120−150 µA compared to 50 µA in Figure 4D_2_, that is, they are all much higher than that of the bare GCE in Figure 5A (15 µA). 

The *I*_pa_ of Au/GCEs reached the maximum value at pH 7; only the AuNF/GCE and the 2D-AuNF/GCE had some slight perturbation. A pH value of 7 is ideal for the determination of NO_2_^−^ because NO_2_^−^ is unstable and easy to decompose in acidic solution, while it is easily reduced to NH_3_ in alkaline solution [37]. The experimental results indicate that these Au/GCEs can increase the electron transfer rate and improve the electrochemical activity of the electrode surface, and these Au/GCEs modified by Au nanoparticles in Figure 4A_1_–C_1_ are more adaptable to the electrocatalytic oxidation and electrochemical detection of nitrites. Hence, pH 7 was selected as one of the appropriate oxidation conditions because all *I*_pa_ in Figure 4 reached the highest value at pH 7. Furthermore, the redox potential differences in Figure 4A_2_–C_2_ are 0.285 V, 0.310 V, and 0.292 V at pH 7, indicating a slight decrease compared with the bare GCE (0.310 V), meaning that the reversibility of Au/GCEs is slightly improved.

#### 2.3.2. The Surface Area of Electrodes

The surface area of electrodes can be indicated by the anodic peak current *I*_pa_ since it is proportional to the *I*_pa_ (Equation (4)) [21]; therefore, the surface area change of Au/GCEs was investigated according to the Randles–Ševčík equation (Equation (4)).

(4) $$I_{\mathrm{pa}}=\;2.69\;\times 10^{-5}\times n^{3/2}\times A\times D^{1/2}\times v^{1/2}\times c$$

where *I*_pa_ is the anodic peak current (μA), *n* is the number of electrons transferred, *A* is the surface area of electrode (cm^2^), *D* is the diffusion coefficient of the detected molecules (cm^2^·s^−1^), *v* is the scan rate (V·s^−1^), and *c* is the concentration of substance detected (mmol·L^−1^).

As shown in Equation (5), the ratio of the surface area of Au/GCEs to that of GCE (*A*_Au/GCE_/*A*_GCE_) is equal to the ratio of the *I*_pa_ of Au/GCE to that of GCE.

(5) $$\frac{A_{\mathrm{Au}/\mathrm{GCE}}}{A_{\mathrm{GCE}}}=\frac{I_{\mathrm{pa},\;\mathrm{Au}/\mathrm{GCE}}}{I_{\mathrm{pa},\;\mathrm{GCE}}}$$

Figure 5B shows the CV curves of the solution of K_3_[Fe(CN)_6_] and KCl detected with different Au/GCEs. Compared with the bare GCE, all Au/GCEs significantly reduced the redox potential difference of K_3_[Fe(CN)_6_]. The *I*_pa_ values of the bare GCE, the AuND/GCE, the AuNF/GCE, the 2D-AuNF/GCE, and the AuNP/GCE are 32.8 µA, 57.5 µA, 60.3 μA, 57.5 µA, and 49.2 µA, respectively. Substituting the *I*_pa, Au/GCE_ and *I*_pa, GCE_ values into Equation (5), the corresponding *A*_Au/GCE_/*A*_GCE_ values are 1.753, 1.838, 1.753, and 1.500, respectively, indicating that the AuNF/GCE has the largest *A*_Au/GCE_/*A*_GCE_ to provide the most catalytic sites for nitrite oxidation.

#### 2.3.3. The Influence of the Scan Rate on Electrochemical Behavior

**Figure 6:**
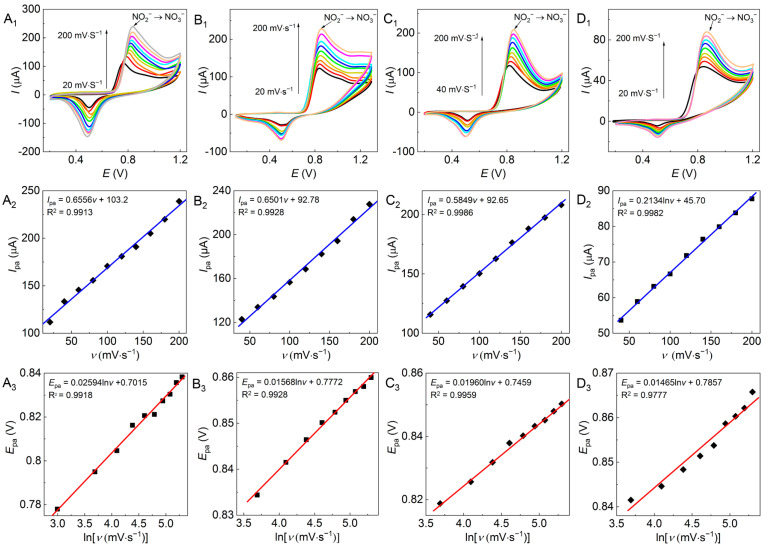
CV plots (**A_1_**–**D_1_**), *I*_pa_–*v* (**A_2_**–**D_2_**), and *E*_pa_–ln*v* (**A_3_**–**D_3_**) referring to the electrocatalytic oxidation of nitrites (5 mmol·L^−1^) detected by various Au/GCEs as (**A**) the AuND/GCE, (**B**) the AuNF/GCE, (**C**) the 2D-AuNF/GCE, and (**D**) the AuNP/GCE at pH 7 with a scan rate range of 20–200 mV∙s^−1^ and 10 scanning turns.

According to the Laviron theory equation [38] (Equation (6)), a higher scan rate would yield a higher oxidation peak potential *E*_pa_. The influence of the scan rate on electrochemical behavior (Figure 6A_1_–D_1_) was investigated with the four Au/GCEs (Figure 4A_1_−D_1_) at pH 7 with a scan rate range of 20–200 mV∙s^−1^.

(6) $$E_{\mathrm{pa}}=E^{0\prime }+\frac{RT}{\alpha nF}\ln\frac{RTk^{0}}{\alpha nF}+\frac{RT}{\alpha nF}\ln v$$

where *E*_pa_ is the oxidation peak potential (V), *E*^0^′ is the standard potential (V), *R* is the ideal gas constant (8.314 J·mol^−1^·K^−1^), *F* is the Faraday constant (96,485 C∙mol^−1^), *α* is the electron transfer coefficient, *k*^0^ is the standard rate constant, *n* is the electron transfer number (2), and *v* is the scan rate (V·s^−1^).

As shown in Figure 6, all assayed Au/GCEs presented an effective electrochemical response on NO_2_^−^. The *I*_pa_ increased linearly with the increase in the scan rate (Figure 6A_2_–D_2_), indicating that the electron transfer of NO_2_^−^ to NO_3_^−^ on these Au/GCEs was controlled by the adsorption step [39]. Meanwhile, ln*v* presented a linear relation with *E*_pa_ (Figure 6A_3_–D_3_), which is consistent with the literature [8].

The corresponding electron transfer coefficients were obtained by substituting the electron transfer number (2) of nitrite oxidation into Equation (6), and the corresponding *α* values of the four Au/GCEs (Figure 4A_1_–D_1_) were 0.495, 0.819, 0.655, and 0.877, respectively. Except for the dendritic Au nanoparticles in Figure 4A_1_, the remainder were all higher than 0.5, meaning these morphologies of Au nanoparticles corresponding to Figure 4B_1_–D_1_ can accelerate the oxidation rate of NO_2_^−^ [19,40] and thus improve the electrochemical activity of these Au/GCEs. Nevertheless, the higher current signal of the dendritic Au nanoparticles may be attributed to the fact that more catalytic active sites were provided by the dendritic shape.

#### 2.3.4. The Limit of Detection of NO_2_^−^ Detected by the Au/GCEs

**Figure 7:**
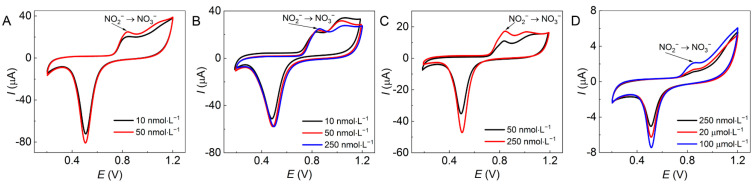
CV plots of the electrocatalytic oxidation of nitrites at low *c*(NO_2_^−^) detected by various Au/GCEs as (**A**) the AuND/GCE, (**B**) the AuNF/GCE, (**C**) the 2D-AuNF/GCE, and (**D**) the AuNP/GCE at pH 7 with a scan rate of 50 mV∙s^−1^ and 10 scanning turns.

The limit of detection (LOD) of NO_2_^−^ was detected by these Au/GCEs using the CV approach, as shown in Figure 7. The lowest *c*(NO_2_^−^) detected by the four Au/GCEs in Figure 4A_1_–D_1_ are 0.01 µmol·L^−1^, 0.01 µmol·L^−1^, 0.05 µmol·L^−1^, and 100 µmol·L^−1^, corresponding to *I*_pa_ values of 20.3 µA, 22.2 µA, 10.9 µA, and 2.1 µA, respectively. The experimental results show that these Au/GCEs possess higher sensitivity for nitrite detection than unmodified GAE; the GCE modified by AuNFs (Figure 4B_1_) provided the strongest catalytic activity and greatest conductivity for nitrite oxidation, followed by the dendritic Au nanoparticles (Figure 4A_1_) and the 2D AuNFs (Figure 4C_1_), whilst the Au nanoplates (Figure 4D_1_) were the worst.

## 3. Discussion

### 3.1. The Selection of the Optimal Electrode

**Figure 8:**
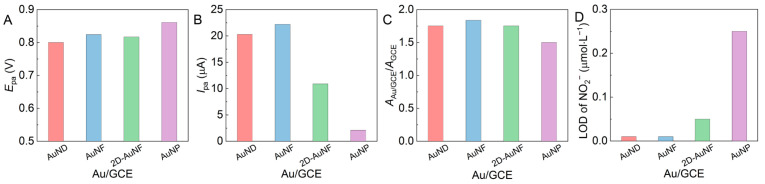
(**A**) *E*_pa_ and (**B**) *I*_pa_ values of four Au/GCEs that detected nitrites (5 mmol·L^−1^) at pH 7, (**C**) *A*_Au/GCE_/*A*_GCE,_ and (**D**) the LOD of NO_2_^−^ detected by these Au/GCEs.

The preferred electrode was screened out by comparing the electrochemical parameters of the four Au/GCEs in Figure 8.

As shown in Figure 8A, the AuNP/GCE presented the undesired highest *E*_pa_ value, and the others exhibited similar *E*_pa_ values at 5 mmol·L^−1^ of sodium nitrite and pH 7. Meanwhile, the AuNP/GCE also yielded the undesired lowest *I*_pa_ value while the AuNF/GCE resulted in the highest *I*_pa_ value (Figure 8B). Figure 8C illustrates that the specific surface areas of all Au/GCEs (*A*_Au/GCE_/*A*_GCE_) were larger than that of bare GCE, and the AuNF/GCE provided the largest (1.838-fold that of GAE), which means that the AuNF/GCE may have the most catalytic active sites or the strongest adsorption capacity and conductivity. Therefore, both the AuNF/GCE and the AuND/GCE (Figure 8D) had the desired lowest detection limit of *c*(NO_2_^−^) (0.01 mmol·L^−1^). The experimental results prove that Au nanoparticles with rich nano-protrusion and a smaller particle size are conducive to the increase in the specific surface areas of Au/GCEs. 

In summary, the AuNF/GCE has the largest specific surface area (1.838-fold that of ACE), providing the most catalytic active sites and the lowest detection limit of *c*(NO_2_^−^) (0.01 µmol·L^−1^), so the AuNF/GCE is the best candidate for Au/GCEs to construct the optimal electrochemical sensing platform for the subsequent nitrite detection in this study. 

### 3.2. The Linear Range and Reproducibility of the AuNF/GCE and Interference for Electrocatalytic Nitrite Oxidation

**Figure 9:**
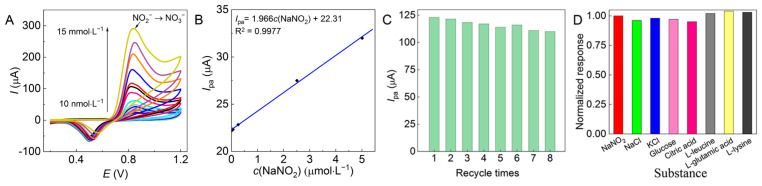
(**A**) CV plots and (**B**) *I*_pa_–*c*(NO_2_^−^) of the AuNF/GCE in electrocatalytic oxidation of nitrites at different *c*(NO_2_^−^) values of 0.01–5.00 µmol·L^−1^ and pH 7 with a scan rate of 50 mV∙s^−1^ and 10 scanning turns, and (**C**) repeatability of and (**D**) interference of the optimal AuNF/GCE.

The correlation between *c*(NO_2_^−^) and *I*_pa_ at pH 7 was investigated with the AuNF/GCE as shown in Figure 9A,B. The electrocatalytic oxidation of nitrites detected by the AuNF/GCE shows a linear relationship between *I*_pa_ and *c*(NO_2_^−^) in the range of 0.01–5.00 µmol·L^−1^, and the calculated sensitivity is 1.966 μA·L·μmol^−1^. The relative results obtained from other reported Au-modified electrodes using either the same CV method or other detection techniques such as differential pulse voltammetry (DPV) are compared in Table 1. The AuNF/GCE demonstrated the lowest detection limit of NO_2_^−^ in Table 1; thus, the higher sensitivity maintains the linear detectable *c*(NO_2_^−^) range within two orders of magnitude in such a low range of *c*(NO_2_^−^), which can provide high operational flexibility for sample pretreatment and preparation. 

As shown in Figure 9C, the electrocatalytic oxidation of nitrites was determined repeatedly eight times on a single AuNF/GCE with 10 scanning turns each time, and the corresponding *I*_pa_ decreased slowly within 4.0%, showing good stability of the AuNF/GCE. Furthermore, the anti-interference of the AuNF/GCE in nitrite detection was investigated under interference from NaCl and KCl (100 times) or glucose, citric acid, L-leucine, L-glutamic acid, and L-lysine (10 times) as high as the tested *c*(NO_2_^−^). The *I*_pa_ values of nitrite solutions with or without interfering substances were obtained from the corresponding CV curves and processed with normalization, and the corresponding normalized response values were obtained as the ratios. The closer these ratios are to 1, the stronger the anti-interference of the modified electrode. The experimental results from Figure 9D show that the above interfering substances did not cause significant interference to the results detected by the AuNF/GCE, indicating that the AuNF/GCE has strong anti-interference.

### 3.3. The Possible Mechanism of Nitrite Detection with the AuNF/GCE

**Scheme 1:**
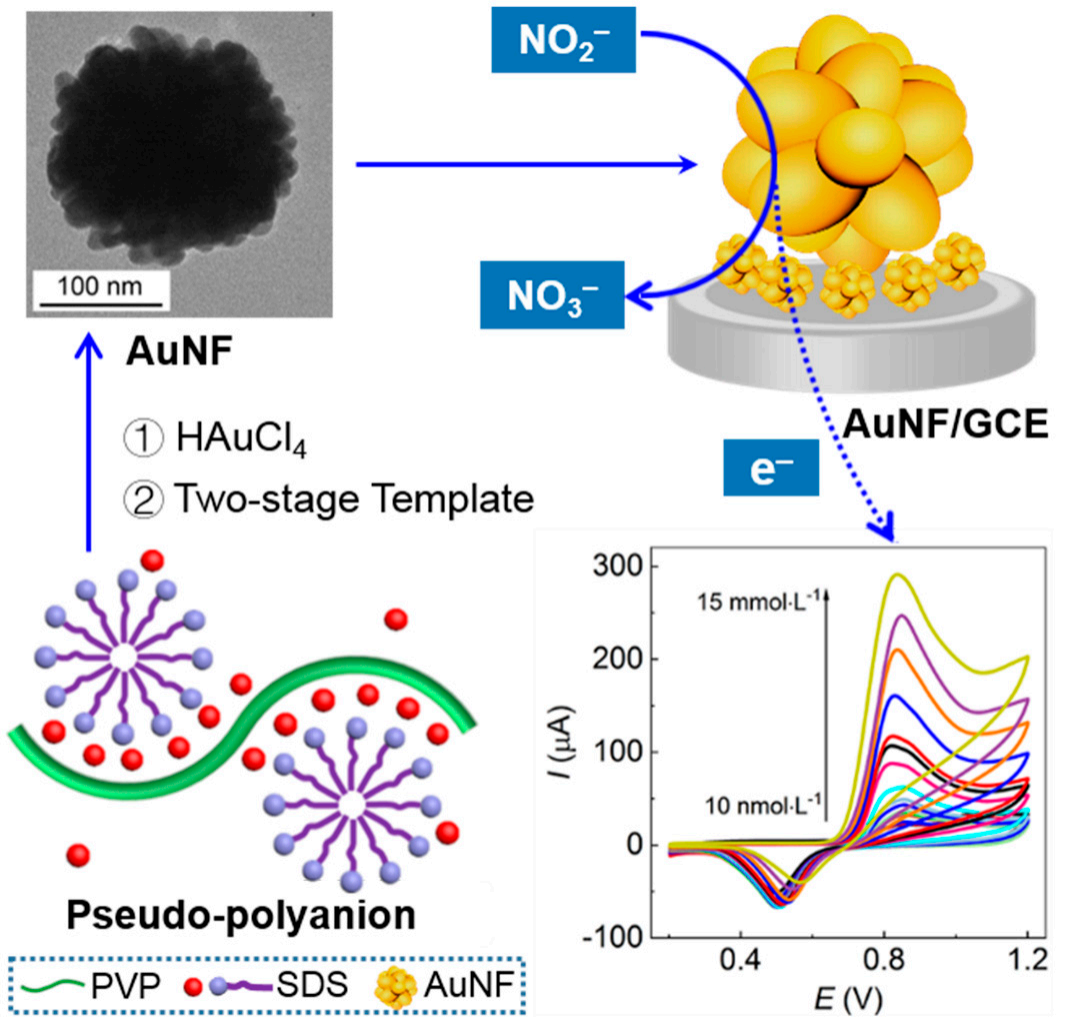
Schematic mechanism of nitrite detection with the optimal AuNF/GCE synthesized by the PVP–SDS pseudo-polyanions with a two-stage soft template function.

The AuNF/GCE presented potential in the sensing of NO_2_^−^ with feasibility and practicability. The mechanism in preparation of the AuNF and AuNF/GCE and the electrocatalytic oxidation and detection of NO_2_^−^ by the AuNF/GCE are proposed in Scheme 1, illustrating the advantages of the greenness and safety, high sensitivity, flexible operation, and feasibility of the electrochemical sensing platform relying on the excellent performance of the AuNF/GCE.

## 4. Materials and Methods

### 4.1. Materials

PVP (K30, MW = 40,000, 99%) was purchased from International Specialty Products Inc. SDS (99%) was purchased from Acros Organics. Chloroauric acid (HAuCl_4_ ≥ 99.9%), sodium nitrite (AR), and other reagents (AR) were purchased from China Sinopharm Co., Ltd, Shanghai, China. Ultrapure Millipore water (18.2 MΩ·cm) was used throughout the experiments.

### 4.2. Synthesis of Au Nanoparticles

PVP and SDS solutions were mixed well in a 20 mL glass reactor and shaken in a water bath at 40 °C for 30 min, followed by the addition of HAuCl_4_ and NaOH successively, and vortexed well. Then, the above mixture was shaken at 40 °C in the dark for another 60 min. The resultant Au nanoparticles were separated by centrifugation at 12,000 rpm (TG-16, Lu Xiangyi Centrifuge Instrument Co., Ltd., Shanghai, China), dispersed in water to form colloidal gold, and stored in the dark for later use. The final concentrations of PVP, SDS, HAuCl_4_, and NaOH in the above mixtures were in the following ranges: *c*(PVP), 5–100 g·L^−1^; *c*(SDS), 0–60 mmol·L^−1^; *c*(HAuCl_4_), 0.25 mmol·L^−1^; and *c*(NaOH), 2 mmol·L^−1^.

### 4.3. Characterization of Au Nanoparticles

The morphology and lattice spacing of the Au nanoparticles were imaged by transmission electron microscopy (TEM, JEM-2100, JEOL, Tokyo, Japan) with an accelerating voltage of 200 kV.

The crystalline phases of the Au nanoparticles were characterized by X-ray diffraction (XRD, D8Advance, Bruker, Karlsruhe, Germany) with a conventional *θ*–2*θ* goniometer using Cu *Kα* radiation (*λ* = 0.15406 nm, scan rate = 4°·min^−1^, and scan 2*θ* range = 20–90°).

The local surface plasmon resonance (LSPR) characteristic peaks of the Au nanoparticles were determined with ultraviolet spectrophotometry (UV-Vis, TU1950, Beijing Purkinje General Instrument Co., Ltd., Beijing, China).

### 4.4. The Electrochemical Behavior of Nitrites Detected by Au/GCEs

The electrochemical activity of the samples was investigated with an electrochemical workstation (CS350, Wuhan Corrtest Instruments Co., Ltd., Wuhan, China) with a three-electrode system that included an opposite electrode (platinum wire), a reference electrode (saturated calomel electrode), and a working electrode (GCE); the GCE was modified by Au nanoparticles (Au/GCEs) to construct the electrochemical sensing platform for nitrite detection. The platinum electrode and the bare GCE were purchased from Tianjin Aida Hengsheng Technology Development Co., Ltd., Tianjin, China, and the calomel electrode was purchased from INESA in China. The preparation of Au/GCEs was as follows: first, the bare GCE with a diameter of 3.0 mm was polished in the Al_2_O_3_ slurry with a diameter of 0.3–0.7 μm, and then, the polished GCE was cleaned with ethanol, ultrapure water, and ethanol alternately under ultrasound for 5 min. After the cleaned GCE was dried by nitrogen blowing, it was placed in 0.1 mol·L^−1^ of K_3_[Fe(CN)_6_] and 0.1 mol·L^−1^ of H_2_SO_4_ solution for 200 consecutive cycles by CV to activate the GCE surface. The above colloidal Au (10 µL) was dropped onto the activated GCE surface and dried naturally for 8–12 h to obtain Au/GCEs modified with various Au nanoparticles. All solutions for electrochemical detection were prepared with 0.1 mol·L^−1^ phosphate buffer solution.

The electrochemical behavior of NO_2_^−^ was investigated by the CV approach to select the optimal Au/GCE; the initial potential was 1.0 V, the termination potential was 0.1 or 0.2 V, and 10 cycles were used.

The reproducibility, repeatability, and stability of the optimal Au/GCE were tested under the above-mentioned conditions.

The influence of inorganics (0.5 mol·L^−1^ of NaCl and KCl) and organics (0.05 mol·L^−1^ of glucose, citric acid, L-leucine, L-glutamic acid, and L-lysine) on the electrochemical behavior of NO_2_^−^ was investigated to verify the interference and stability of the optimal Au/GCE.

## 5. Conclusions

Au nanoparticles were synthesized in the pseudo-polyanions of PVP–SDS by dint of the in situ reduction of HAuCl_4_ with PVP. The experimental results reveal that the particle sizes and morphologies of the Au nanoparticles at room temperature can be regulated by choosing the concentrations of PVP and SDS, in which the PVP-bound SDS micelles may serve as the primary template to confine the primary Au crystals. PVP served as the in situ reductant in the primary template and also as the secondary template to balance the reduction rate of HAuCl_4_ and the stacking degree of the primary Au crystals. Therefore, four types of Au nanoparticles with nanodendritic, nanoflower, 2D nanoflower, and nanoplate shapes were obtained, among which the Au nanoflowers (AuNFs) had an average equivalent particle size range of 100–200 nm with 16–20 nm of rich protrusion on the surface.

Furthermore, the above four Au nanoparticles were applied to modify the bare GCE to obtain different Au/GCEs to construct electrochemical sensing platforms for nitrite detection. The cyclic voltammetry (CV) measurement demonstrated that the four Au/GCEs exhibited a sharp oxidation peak with a narrow oxidation peak potential (*E*_pa_) within 0.800–0.820 V and *I*_pa_ values as high as 50–150 µA, indicating that these four Au nanoparticles endowed the Au/GCEs with improved electrochemical activity in comparison to the bare GCE. The GCE modified by a typical AuNF (the AuNF/GCE) provided the largest *A*_AuNF/GCE_/*A*_GCE_ of 1.838 and the lowest detectable *c*(NO_2_^−^) of 0.01 µmol·L^−1^ with an *I*_pa_ of 22.2 µA, followed by the AuND/GCE. This reveals the morphological effects of the Au nanoparticles on the electrochemical sensing platforms for nitrite detection, that is, the Au nanoparticle with rich nano-protrusions and smaller size is conducive to the increase in the Au/GCE surface area, providing abundant catalytic activity sites to enhance the detection sensitivity and further upgrade the LOD.

Compared with other previously reported Au-modified electrodes, the AuNF/GCE presented the lowest LOD and the highest sensitivity in the low linear *c*(NO_2_^−^) detection range of 0.01–5 µmol·L^−1^. The AuNF/GCE also displayed great anti-interference ability against some concentrated inorganic and organic interference substances together with good reproducibility, showing high operational flexibility for sample pretreatment and preparation, as well as feasibility in the application of the electrochemical sensing platforms.

## Figures and Tables

**Table 1:** Comparison of different Au/GCEs for nitrite detection.

Au-Modified Electrode	Technique	Linear Range (μmol·L^−1^)	Limit of Detection (μmol·L^−1^)	Sensitivity (μA·L·μmol^−1^)
Au WNWs/CNFs-Gr [8]	Amperometric	1.98–3771	1.24	–
GNPs/UiO-66-NH_2_/Rgo [12]	DPV	5.0–768	3.7	–
Au/CNHN/GCE [13]	Chronoamperometry	0.05–1150	0.017	0.00469
AuNP/PCG/FTO [19]	DPV	0.095–200	0.095	0.063
AuNC@Cu_2_O [23]	Amperometric	0.4–806.6	0.015	18.19
Au-PPy-C/g-C_3_N_4_ NCs/GCE [24]	DPV	1.5–22.5	1.11	2.8816
Au/GCE [41]	CV	5–500	0.5	–
MoS_2_/Au/CM/PGE [42]	DPV	20–350	0.022	0.0216
GONRs-AuNPs/GCE [43]	CV	10–10,000	1.3	0.00775
Au_4.5_NPs@MoS_2_/rGO/GCE [16]	Amperometric	0.2–2600	0.038	0.158
Au/polyaniline/carbon paste electrode [44]	CV	38–1000	25	–
AuNF/GCE (this work)	CV	0.01–5.00	0.01	1.966

## Data Availability

Not applicable.

## Supplementary Materials

The following supporting information can be downloaded at https://www.mdpi.com/article/10.3390/molecules28134934/s1, Figure S1: TEM of Au nanoplates growing in PVP (50 g·L^−1^)–SDS (5 mmol·L^−1^)–HAuCl_4_ (1.0 mmol·L^−1^) solution; Figure S2; LSPR spectra of Au nanoparticles growing in PVP (50 g·L^−1^)–SDS–HAuCl_4_ (0.25 mmol·L^−1^) solutions with different SDS concentrations; Figure S3: LSPR spectra of Au nanoparticles growing in PVP–SDS (5 mmol·L^−1^)–HAuCl_4_ (0.25 mmol·L^−1^) solutions with different PVP concentrations.

## Sample Availability

Samples of the compounds are not available from the authors.
