# Supplementary material for: Morphological Effects of Au Nanoparticles on Electrochemical Sensing Platforms for Nitrite Detection
Source: Molecules. 2023 Jun 23;28(13):4934. doi: 10.3390/molecules28134934 (PMC10343565; doi:10.3390/molecules28134934)
Supplement: Supplementary file 1 [file molecules-28-04934-s001.zip › molecules-2424294-supplementary.pdf]

Supporting Materials

Morphological Effects of Au Nanoparticles on Electrochemical Sensing Platforms for Nitrite Detection

Ruiqin Feng [1], Ye Fan [1], Yun Fang [1,*], Yongmei Xia [1,2,*]

[1]*The Key Laboratory of Synthetic and Biological Colloids (Ministry of Education), School of Chemical and Material Engineering, Jiangnan University, Wuxi, Jiangsu 214122, P. R. China*

[2]*State Key Laboratory of Food Science and Technology, Jiangnan University, Wuxi, Jiangsu 214122, P. R. China*

Caption list

Figure S1. TEM of Au nanoplates growing in PVP(50 g·L^{-1})–SDS(5 mmol·L^{-1})–$HAuCl_4$(1.0 mmol·L^{-1}) solution.

Figure S2. LSPR spectra of Au nanoparticles growing in PVP(50 g·L^{-1})–SDS–$HAuCl_4$(0.25 mmol·L^{-1}) solutions with different SDS concentrations.

Figure S3. LSPR spectra of Au nanoparticles growing in PVP–SDS(5 mmol·L^{-1})–$HAuCl_4$(0.25 mmol·L^{-1}) solutions with different PVP concentrations.

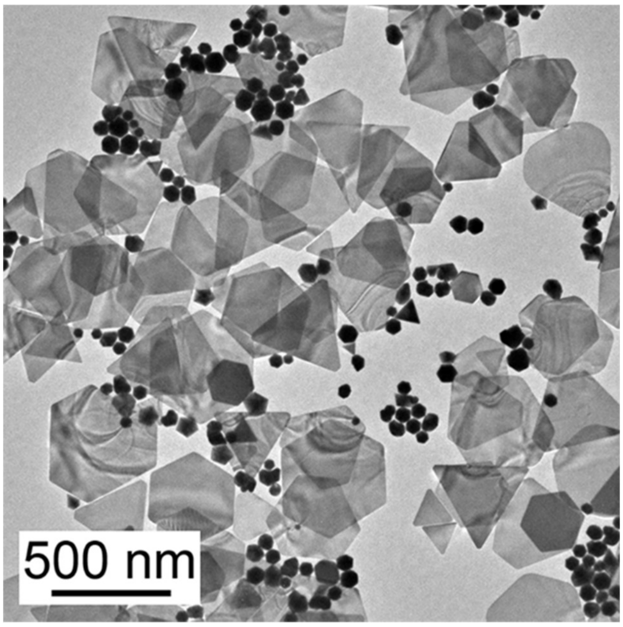

Figure S1. TEM of Au nanoplates growing in PVP(50 g·L^{-1})–SDS(5 mmol·L^{-1})–$HAuCl_4$(1.0 mmol·L^{-1}) solution.

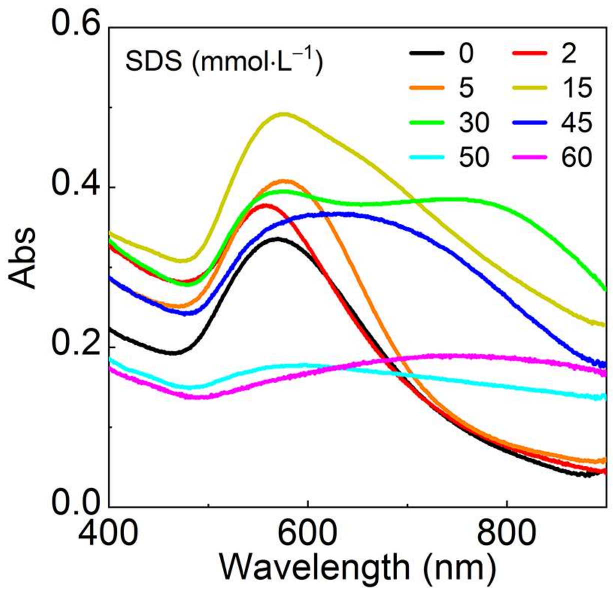

Figure S2. LSPR spectra of Au nanoparticles growing in PVP(50 g·L^{-1})–SDS–$HAuCl_4$(0.25 mmol·L^{-1}) solutions with different SDS concentrations.

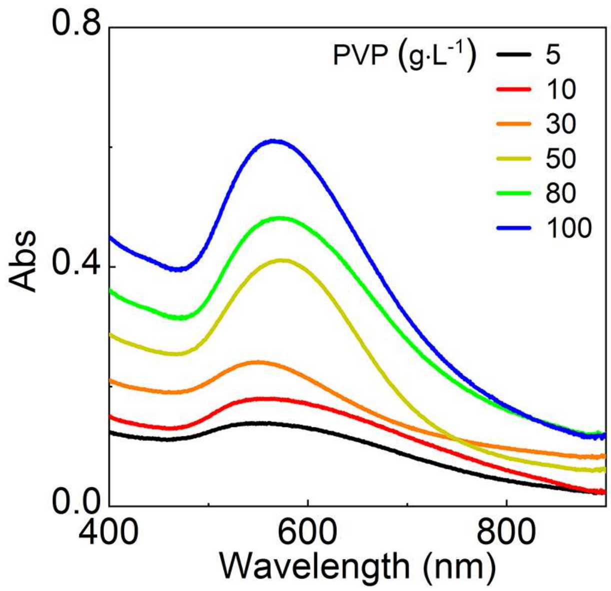

Figure S3. LSPR spectra of Au nanoparticles growing in PVP–SDS(5 mmol·L^{-1})–$HAuCl_4$(0.25 mmol·L^{-1}) solutions with different PVP concentrations.
